# Unintended right atrial lesions after monopolar biphasic lattice-tip pulsed-field ablation: a case series

**DOI:** 10.1093/ehjcr/ytag730

**Published:** 2026-09-30

**Authors:** Jun Hirokami, Kenichi Hiroshima, Michio Nagashima, Masato Fukunaga, Kengo Korai, Kenji Ando

**Affiliations:** Department of Cardiology, Kokura Memorial Hospital, 1-2-3 Asano, North of Kokura, Kitakyushu, Fukuoka 802-8555, Japan; Department of Cardiology, Kokura Memorial Hospital, 1-2-3 Asano, North of Kokura, Kitakyushu, Fukuoka 802-8555, Japan; Department of Cardiology, Kokura Memorial Hospital, 1-2-3 Asano, North of Kokura, Kitakyushu, Fukuoka 802-8555, Japan; Department of Cardiology, Kokura Memorial Hospital, 1-2-3 Asano, North of Kokura, Kitakyushu, Fukuoka 802-8555, Japan; Department of Cardiology, Kokura Memorial Hospital, 1-2-3 Asano, North of Kokura, Kitakyushu, Fukuoka 802-8555, Japan; Department of Cardiology, Kokura Memorial Hospital, 1-2-3 Asano, North of Kokura, Kitakyushu, Fukuoka 802-8555, Japan

**Keywords:** Atrial fibrillation, Pulsed-field ablation, A lattice-tip catheter, Deep sedation, Pulmonary vein isolation, Unintentional lesion

## Abstract

**Background:**

Monopolar biphasic pulsed-field ablation (PFA) delivered by a lattice-tip catheter may create deeper lesions than conventional bipolar systems. The impact on adjacent right atrial structures remains poorly characterized.

**Case summary:**

We report four patients undergoing pulmonary vein isolation using a lattice-tip PFA catheter. Post-procedural electroanatomic mapping revealed unintended right atrial lesions involving the posterior right atrium, interatrial septum, or superior vena cava. In patients with short anatomical distances between right pulmonary veins and adjacent right atrial structures, lesions developed despite apparently successful pulmonary vein isolation. Catheter instability related to coughing under deep sedation appeared to contribute to broader lesion formation in two cases.

**Discussion:**

These cases suggest that monopolar biphasic lattice-tip PFA may create unintended lesions in adjacent right atrial tissue when anatomical proximity is short or catheter stability is compromised. Awareness of these mechanisms may help optimize procedural safety and lesion delivery.

Learning points

- Monopolar biphasic lattice-tip PFA may create unintended lesions beyond the intended pulmonary vein isolation line.
- Short anatomical distance between right pulmonary veins and adjacent right atrial structures appears to increase the likelihood of collateral lesion formation.
- Coughing and catheter instability during deep sedation may contribute to extensive right atrial lesions.

## Introduction

Pulsed-field ablation (PFA) has emerged as an effective treatment strategy for atrial fibrillation (AF) because of its myocardial selectivity and favourable safety profile.^1^ The recently introduced lattice-tip Sphere-9 catheter (Affera, Medtronic) differs from conventional single-shot PFA systems by delivering a monopolar biphasic waveform through a focal lattice-tip electrode.^2^ Experimental studies suggest that monopolar biphasic PFA may create deeper lesions than conventional bipolar biphasic systems, potentially improving lesion durability but also increasing the possibility of unintended injury to adjacent myocardial structures.^3,4^ The right pulmonary veins (PVs) are anatomically adjacent to several right atrial structures, including the posterior wall of the right atrium, the interatrial septum, and the superior vena cava (SVC). Although collateral effects on these structures have been reported with single-shot bipolar PFA systems, the impact of monopolar biphasic lattice-tip PFA on neighbouring right atrial tissue remains poorly understood.

We report four consecutive patients undergoing AF ablation with a lattice-tip PFA catheter in whom post-procedural electroanatomic mapping demonstrated unintended right atrial lesions. These cases highlight the potential roles of anatomical proximity and catheter instability in lesion formation outside the intended ablation target.

## Summary figure

**Figure:**
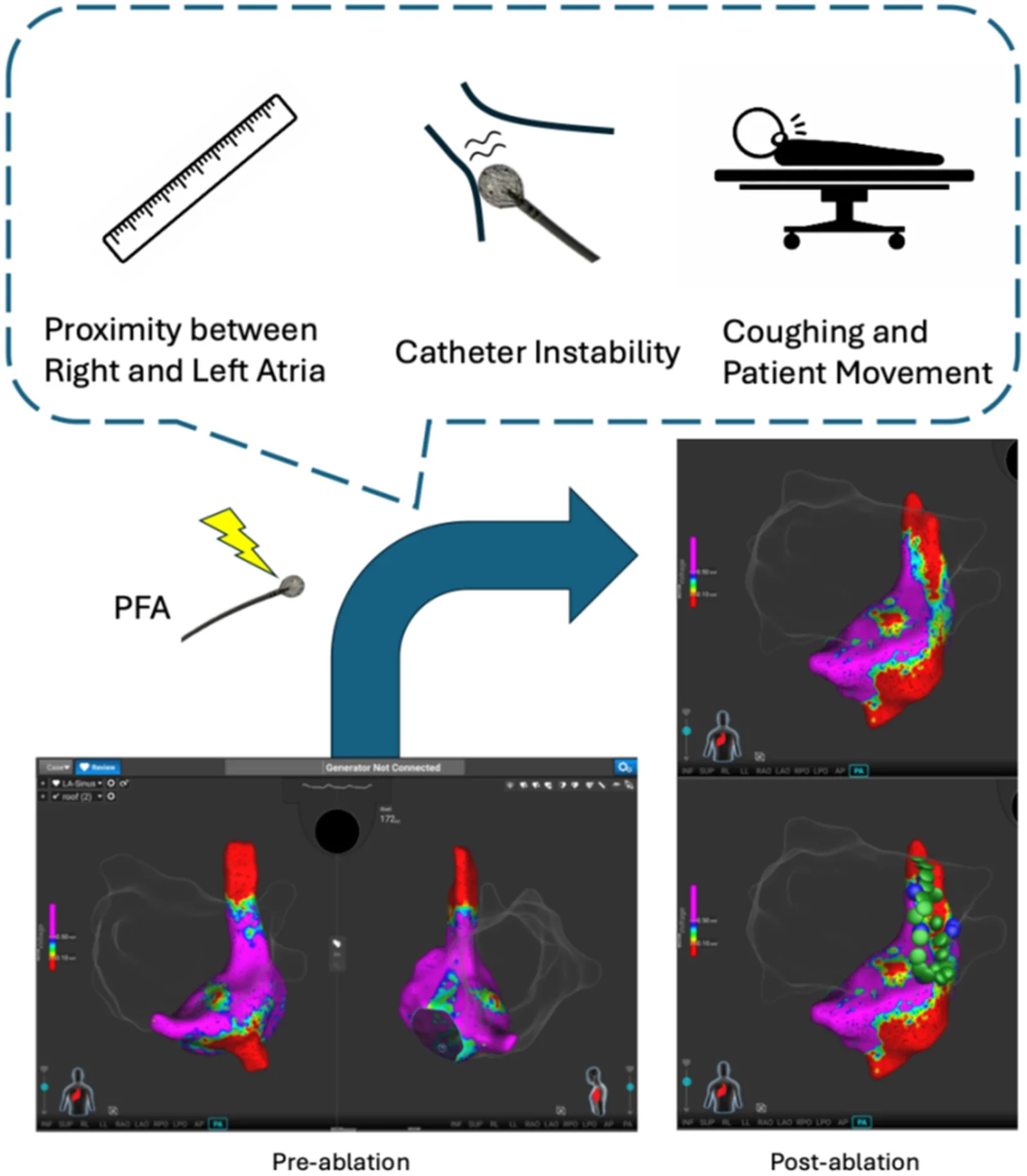
Potential mechanisms of unintended right atrial lesion formation during lattice-tip PFA. Short anatomical distance between right PVs and adjacent right atrial structures, together with catheter instability caused by coughing during deep sedation, may facilitate collateral lesion formation in the right atrium and SVC.

## Ablation procedures

All ablations were performed under deep sedation using propofol, thiopental, and pentazocine. After femoral venous access was obtained, intravenous heparin was administered to maintain an activated clotting time ≥ 350 s. A 20-pole catheter was inserted through the right femoral vein. The proximal electrodes were positioned along the crista terminalis, and the distal electrodes were placed in the coronary sinus. A single transseptal puncture was performed using an 8-Fr sheath and a Brockenbrough needle under fluoroscopic guidance. Both ablation and mapping were performed using a Sphere-9 catheter. Four dispersive return electrode patches were placed on the patient’s back according to the manufacturer-recommended standard configuration of the Affera system, and the same configuration was used in all patients. To evaluate both intended ablation lesions and unintentional lesion formation, three-dimensional mapping of both atria was performed in sinus rhythm before and after ablation. If AF persisted, internal cardioversion was performed to restore sinus rhythm prior to mapping. Bipolar voltage was measured as the peak-to-peak electrogram amplitude during sinus rhythm. A bipolar voltage of <0.1 mV was defined as low voltage, 0.1–0.5 mV as mid voltage, and >0.5 mV as normal voltage. Mapping was performed using the nominal system settings, including respiration gating at 80%, a voltage threshold of 0.05 mV, and an activation threshold of 0.02 V/s. Identical acquisition and voltage display settings were used for the pre- and post-ablation maps in each patient. When map shift due to displacement of the magnetic reference patch was recognized, the map position was manually corrected, and catheter location was anatomically verified using fluoroscopy. After ablation, a new right atrial map was created in each patient, with both the geometry and high-density voltage map reconstructed *de novo* rather than by adding mapping points to the pre-ablation geometry. All ablation applications were delivered exclusively using pulsed-field energy. Pulsed-field ablation was delivered using the nominal PULSE3 setting, a preclinically optimized biphasic monopolar waveform, with a current limit of 95%, a pulse duration of 4 s, and an irrigation flow rate of 15 mL/min.^2,5^ After ablation, high-output pacing at 10 V was performed from the newly identified low-voltage regions to assess local myocardial excitability. No local capture was observed in these regions. Catheter location was assessed using both electroanatomic mapping and fluoroscopic guidance. Catheter-tissue contact was assessed using impedance-based contact-sensing information provided by the Affera system in combination with fluoroscopic visualization of catheter position and movement. After ablation, patients were scheduled for clinical follow-up at 1, 3, 6, and 12 months, with rhythm assessment using a 12-lead electrocardiogram or 1-week Holter monitoring. Unscheduled evaluations were additionally performed if patients developed symptoms suggestive of arrhythmia recurrence.

## Computed tomography assessment of anatomical proximity

Anatomical distances between the right PVs and adjacent right atrial structures were measured on contrast-enhanced computed tomography (CT) images reconstructed at the diastolic phase. The lumen-to-lumen distance was measured primarily on axial images by a single operator.

## Case presentations

All patients provided written informed consent for participation upon admission.

### Patient 1

An 87-year-old female was diagnosed with symptomatic persistent AF, refractory to pharmacological therapy including a β-blocker and amiodarone. She developed congestive heart failure, and rhythm control was deemed necessary. Transthoracic echocardiography (TTE) showed a reduced left ventricular ejection fraction (LVEF) of 35%, a left atrial diameter (LAD) of 36 mm, and a left atrial volume index of 68. Laboratory data were as follows: brain natriuretic peptide (BNP), 228.5 pg/mL; thyroid-stimulating hormone (TSH), 6.44 μIU/mL; free thyroxine, 1.24 ng/dL; blood urea nitrogen (BUN), 35.4 mg/dL; and creatinine, 1.25 mg/dL. Her past medical history was significant for hypertension and dyslipidaemia. Contrast-enhanced CT demonstrated that the distance between the SVC and the RSPV at the pulmonary vein isolation (PVI) line ranged from 1.6 to 1.9 mm.

Using a Sphere-9 catheter, all four PVs were circumferentially ablated with PF energy, achieving bidirectional block (28 applications for the right PVs, 27 for the left PVs). No map shift occurred during PFA due to coughing or patient movement. The pre- and post-ablation RA maps comprised 2731 and 2613 mapping points, respectively. In the RA, a longitudinal band-like unintentional lesion was observed along the PVI line on the posterior wall (*Figure 1*).

**Figure 1:**
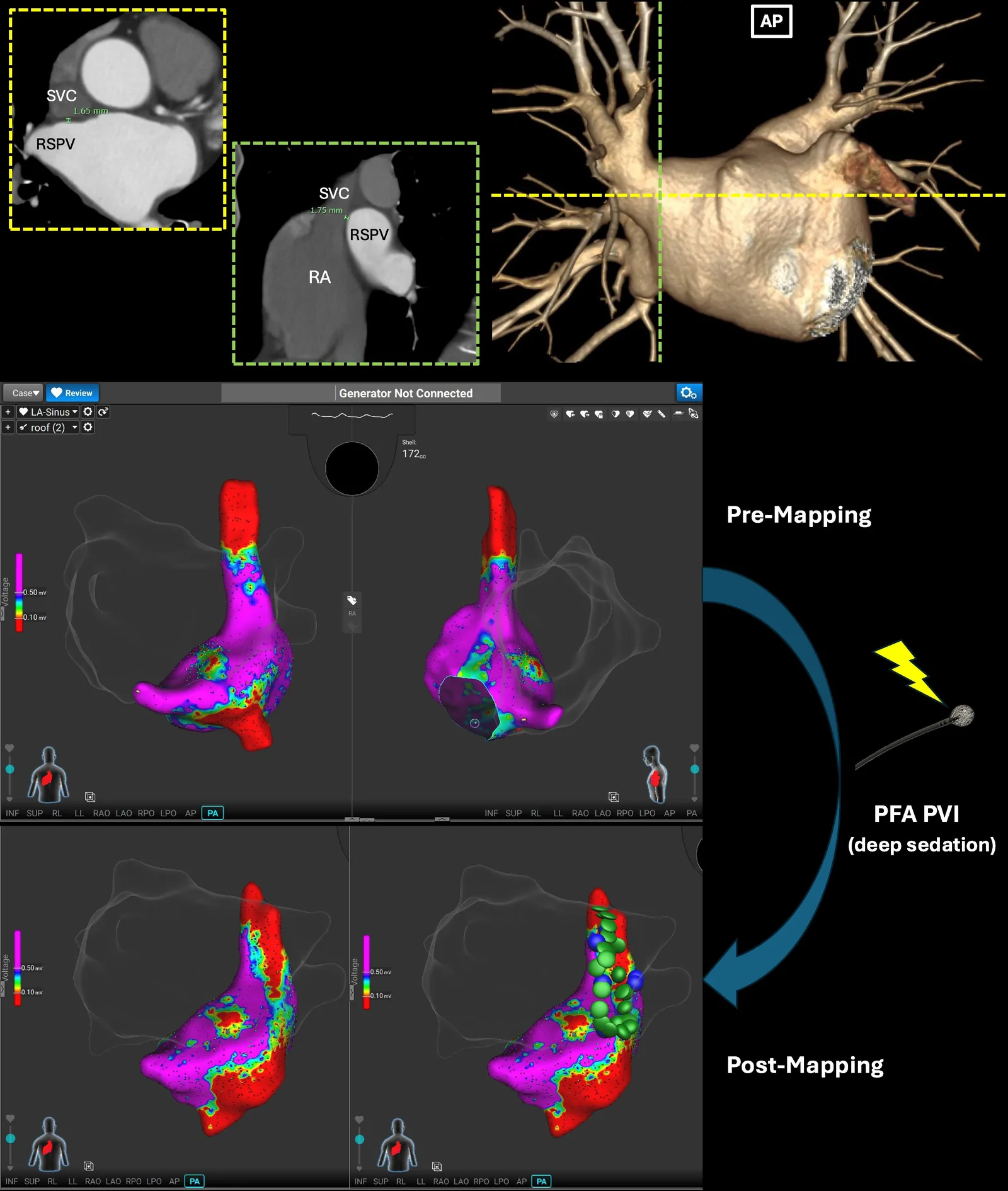
An 87-year-old woman with persistent atrial fibrillation. Yellow dots indicate the axial view and green dots the sagittal view on contrast-enhanced computed tomography. Green and blue tags indicate ablation sites in the left atrium. AP, anteroposterior; PFA, pulsed-field ablation; PVI, pulmonary vein isolation; RA, right atrium; RSPV, right superior pulmonary vein; SVC, superior vena cava.

### Patient 2

A 64-year-old man with symptomatic persistent AF reported exertional dyspnoea and palpitations despite treatment with a β-blocker and flecainide. Therefore, the patient elected to undergo catheter ablation for rhythm control. Transthoracic echocardiography showed a normal LVEF of 55%, an LAD of 51 mm, and a left atrial volume index of 45. Laboratory data were as follows: BNP, 96.1 pg/mL; TSH, 2.52 μIU/mL; free thyroxine, 0.99 ng/dL; BUN, 11.4 mg/dL; and creatinine, 0.90 mg/dL. He had no significant past medical history. Computed tomography demonstrated that although the distance between the right superior pulmonary vein (RSPV) and the SVC was ∼6 mm, it was shorter at the mid-to-lower portion of the PV antrum, ranging from 1.6 to 3.0 mm.

All four PVIs and left atrial posterior wall isolation were achieved using only PF energy (32 applications for the right PVs, 34 for the left PVs, 8 for the roof line, and 15 for the inferior line). During ablation of the right PVs, coughing resulted in an upward shift of the three-dimensional map. Furthermore, the catheter occasionally advanced into the PV in synchrony with coughing during energy delivery. The pre- and post-ablation RA maps comprised 3271 and 2703 mapping points, respectively. In the right atrium (RA), a low-voltage zone was observed extending from the inferior vena cava to the right atrial septum, while a mid-voltage zone was distributed over the posterior wall, corresponding to the region opposite the PVs (*Figure 2*).

**Figure 2:**
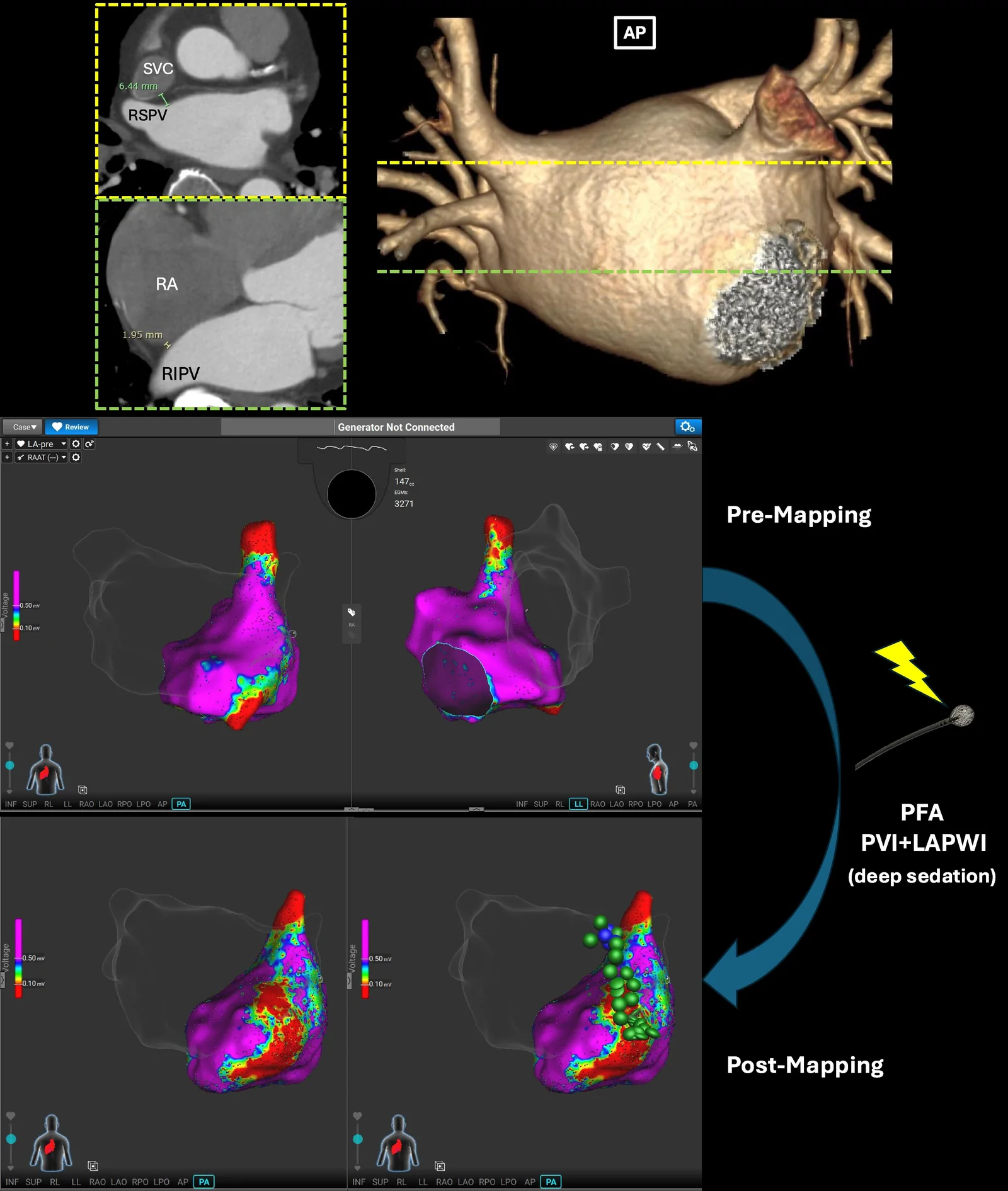
A 64-year-old man with persistent atrial fibrillation. Yellow and green dots indicate the axial view on contrast-enhanced computed tomography. Green and blue tags indicate ablation sites in the left atrium. AP, anteroposterior; LAPWI, left atrial posterior wall isolation; PFA, pulsed-field ablation; PVI, pulmonary vein isolation; RA, right atrium; RSPV, right superior pulmonary vein; SVC, superior vena cava.

### Patient 3

A 77-year-old man with paroxysmal AF reported recurrent palpitations, refractory to treatment with a β-blocker. As the frequency of episodes increased, the patient elected to undergo catheter ablation. Transthoracic echocardiography showed a normal LVEF of 60%, an LAD of 40 mm, and a left atrial volume index of 30. Laboratory data were as follows: BNP, 85.6 pg/mL; TSH, 1.41 μIU/mL; free thyroxine, 1.11 ng/dL; BUN, 14.3 mg/dL; and creatinine, 0.88 mg/dL. His past medical history was significant only for hypertension. Computed tomography demonstrated that the distance between the SVC and the RSPV ranged from 1.3 to 1.8 mm. In contrast, the right inferior pulmonary vein (RIPV), including its antrum, was completely separated, with a minimum distance of 5.7 mm.

Three-dimensional mapping performed after bilateral PVI and left atrial posterior wall isolation demonstrated loss of voltage at the posterior wall of the SVC, which was in close proximity to the RSPV on pre-procedural CT (30 applications for the right PVs, 29 for the left PVs, 6 for the roof line, and 11 for the inferior line) (*Figure 3*). The pre- and post-ablation RA maps comprised 2970 and 2501 mapping points, respectively. No coughing or map shift was observed during ablation in this patient.

**Figure 3:**
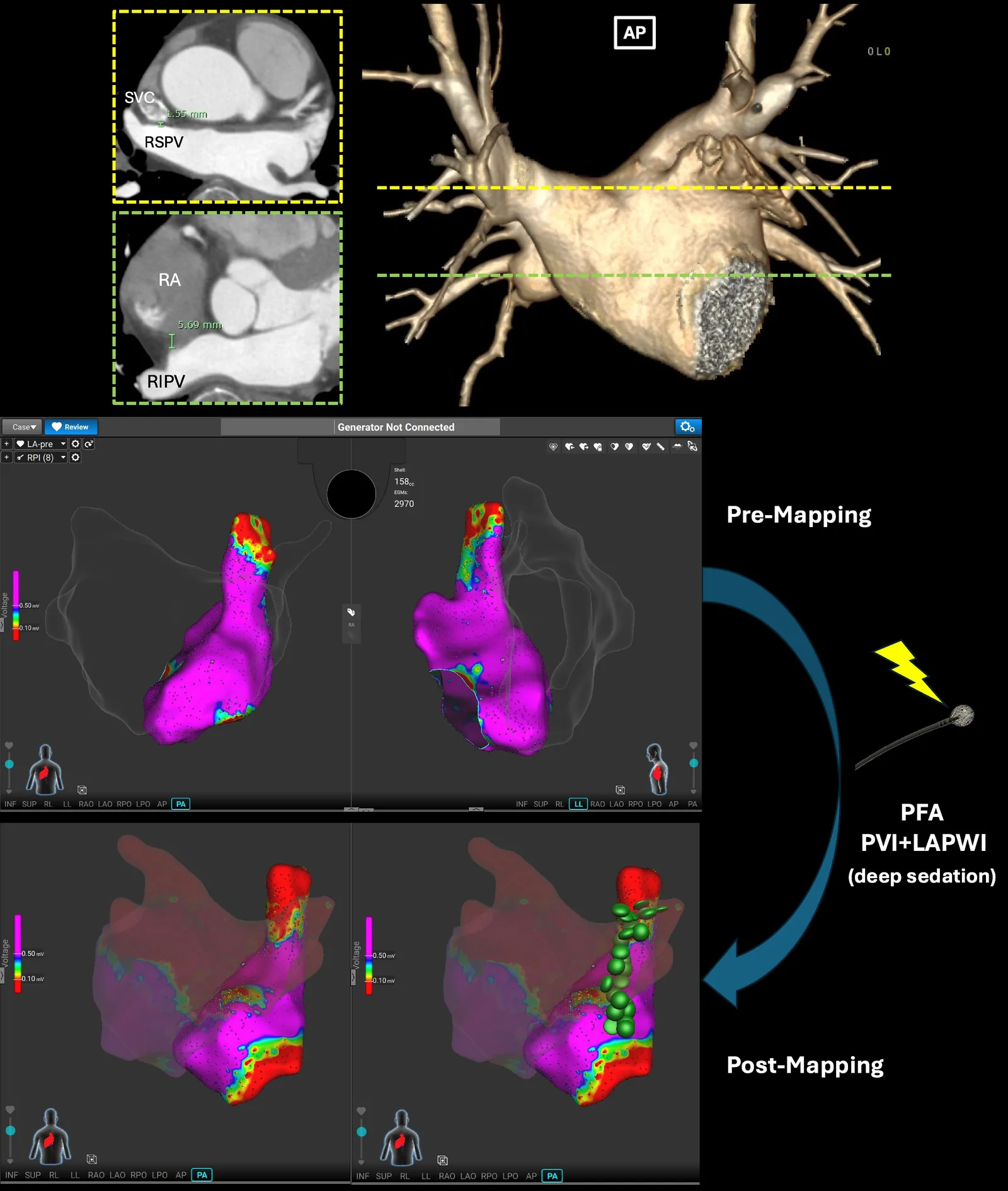
A 77-year-old man with paroxysmal atrial fibrillation. Yellow and green dots indicate the axial view on contrast-enhanced computed tomography. Green tags indicate ablation sites in the left atrium. AP, anteroposterior; LAPWI, left atrial posterior wall isolation; PFA, pulsed-field ablation; PVI, pulmonary vein isolation; RA, right atrium; RIPV, right inferior pulmonary vein; RSPV, right superior pulmonary vein; SVC, superior vena cava.

### Patient 4

A 66-year-old female was admitted due to congestive heart failure with persistent AF and reported exertional dyspnoea and lower extremity oedema lasting for 2 months. Her past medical history was significant only for dyslipidaemia. Following treatment for heart failure, AF persisted despite antiarrhythmic drug therapy, and catheter ablation was subsequently performed. Transthoracic echocardiography showed a mildly reduced LVEF of 51%, an LAD of 40 mm, and a left atrial volume index of 30. Laboratory data were as follows: BNP, 201.2 pg/mL; TSH, 0.95 μIU/mL; free thyroxine, 1.08 ng/dL; BUN, 15.5 mg/dL; and creatinine, 0.73 mg/dL. Computed tomography demonstrated that the distance between the antrum of the RSPV and the SVC was greater than 5 mm. However, the left atrium was enlarged in the longitudinal direction, and the inferior vena cava and the posterior wall of the RA were displaced by the RIPV antrum. As a result, the distance between the RIPV antrum and the posterior wall of the right atrium was ∼2 mm.

Bilateral PVI and left atrial posterior wall isolation were performed (37 applications for the right PVs, 36 for the left PVs, 5 for the roof line, and 6 for the inferior line). The pre- and post-ablation RA maps comprised 3242 and 2912 mapping points, respectively. Marked coughing during each PF energy application led to significant map shift. Furthermore, catheter stability during energy delivery was compromised, resulting in a broad unintentional low-voltage zone extending from the right atrial septum to the posterior wall on post-procedural mapping (*Figure 4*).

**Figure 4:**
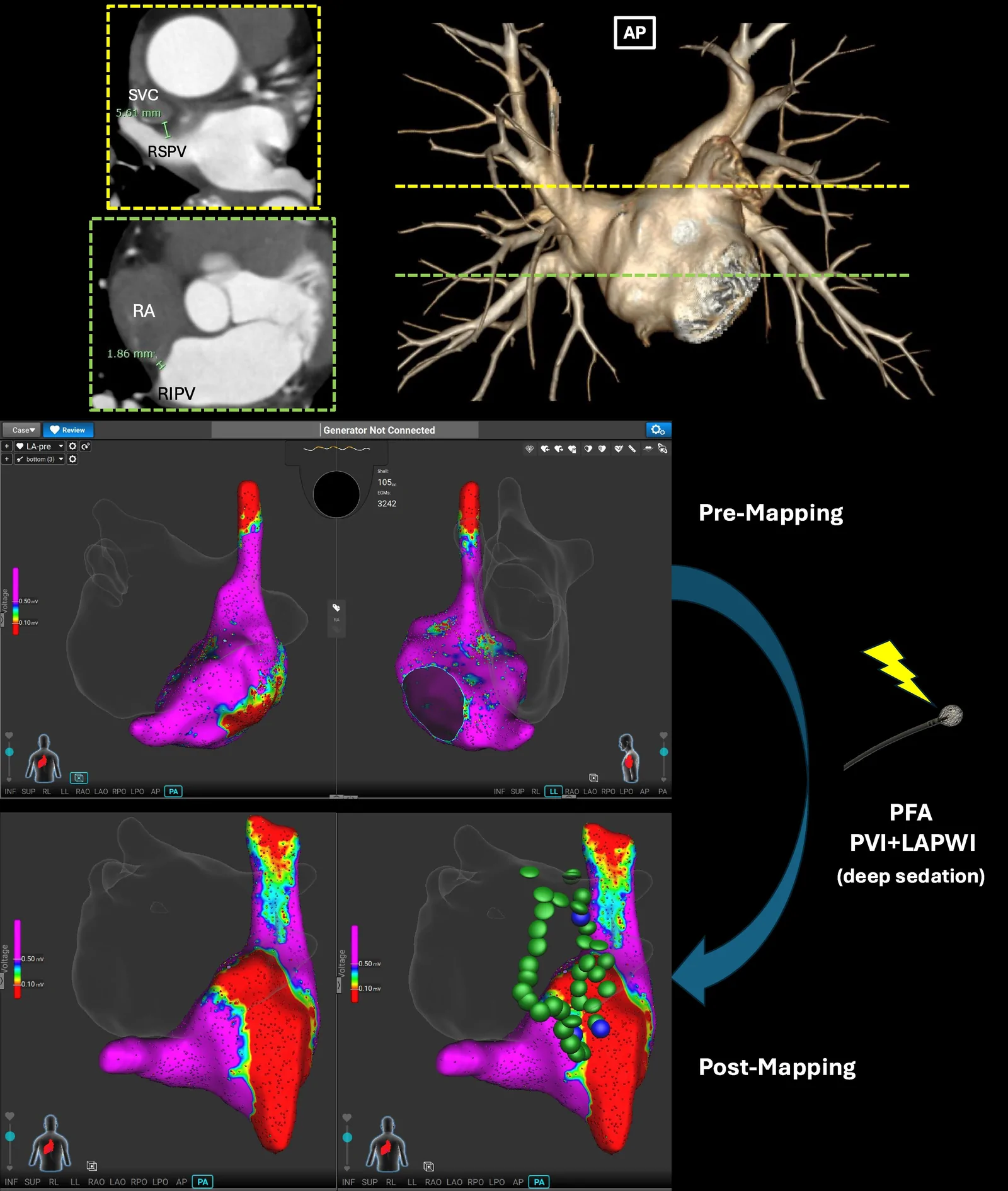
A 66-year-old female with persistent atrial fibrillation. Yellow and green dots indicate the axial view on contrast-enhanced computed tomography. Green and blue tags indicate ablation sites in the left atrium. AP, anteroposterior; LAPWI, left atrial posterior wall isolation; PFA, pulsed-field ablation; PVI, pulmonary vein isolation; RA, right atrium; RSPV, right superior pulmonary vein; SVC, superior vena cava.

## Discussion

This case series describes unintended right atrial lesion formation following PVI using a lattice-tip monopolar biphasic PFA catheter. Unintended lesions were identified in all four consecutive patients treated during our initial experience with this technology. Although the number of cases was limited, the consistent observation suggests that collateral lesion formation in adjacent right atrial structures may represent a clinically relevant phenomenon.

Previous studies using bipolar biphasic single-shot PFA systems have demonstrated unintended effects on neighbouring right atrial structures. Matsuura *et al*. reported that a shorter distance between the right superior PV and the SVC was associated with unintended SVC isolation following PFA.^6^ Similar findings were observed in our series. However, the Sphere-9 system differs from currently available single-shot technologies by delivering monopolar biphasic energy. In bipolar configurations, the electric field is generated primarily between local catheter electrodes, whereas in monopolar configurations, energy is delivered between the ablation electrode and a remote dispersive electrode. Computational modelling and biophysical studies suggest that monopolar delivery may produce a less confined electric field and greater lesion depth than bipolar delivery.^3,7^ Furthermore, computational modelling has demonstrated that electrode configuration and catheter orientation can influence electric field distribution and lesion geometry.^3^ These characteristics may facilitate extension of the electric field beyond the intended target tissue, particularly when the right PVs are in close anatomical proximity to right atrial structures, potentially contributing to the unintended lesions observed in our cases.

Anatomical proximity appeared to be an important determinant of lesion formation. In Patients 2–4, unintended lesions developed in regions where the distance between the right PVs and adjacent right atrial structures was ∼2 mm or less. Experimental studies have shown that monopolar biphasic PFA delivered through a lattice-tip catheter can create relatively deep myocardial lesions. In a histological analysis using a swine model, Kawamura *et al*. reported that lesion depth using a 4-s PF setting originally optimized for atrial ablation was 4.8 mm (interquartile range: 4.5–5.4 mm) in healthy ventricular myocardium.^8^ In atrial myocardium, Yavin *et al*. reported that chronic lesion depth was 1.2 mm (range: 0.4–3.4 mm).^9^ When the interatrial distance is minimal, electric field penetration beyond the intended ablation site may therefore affect neighbouring right atrial tissue.

Catheter instability may represent an additional mechanism. Monopolar PFA is associated with greater skeletal muscle capture than bipolar systems.^10^ Previous studies have demonstrated that ablation using the Sphere-9 catheter under deep sedation is feasible in terms of achieving acute bidirectional block in PVI and linear lesion ablation.^11,12^ However, from the perspective of potential effects on the RA, coughing or patient movement during energy delivery may lead to catheter tip dislodgement. In two patients, coughing during energy delivery resulted in map shifts and apparent catheter displacement. Such movement may transiently reduce the distance between the ablation catheter and adjacent right atrial structures, facilitating unintended lesion creation. The broad septal lesions observed in these patients may have resulted from energy delivery occurring while the catheter was displaced towards the interatrial septum, which is typically only a few millimetres thick. In addition, discrepancies between the electroanatomic map and the actual catheter position cannot be completely excluded and may have contributed to catheter positioning closer to adjacent structures than anticipated.

These findings have potential implications for contemporary AF ablation practice. The current European Society of Cardiology guidelines recognize PFA as an emerging ablation technology with favourable safety characteristics compared with thermal energy sources.^1^ Nevertheless, our observations suggest that collateral myocardial injury within neighbouring cardiac chambers may still occur when monopolar biphasic systems are used in anatomically constrained regions. Careful pre-procedural assessment of cardiac anatomy and attention to catheter stability during energy delivery may help minimize unintended lesion formation.

Several limitations should be acknowledged. First, this report describes only four patients from a single centre and cannot establish the true incidence of unintended right atrial lesions. Second, lesion durability was not assessed because repeat mapping was unavailable. Third, the clinical significance of these lesions remains uncertain, including their impact on atrial arrhythmia recurrence and long-term atrial conduction. Fourth, our observations are specific to a monopolar biphasic lattice-tip catheter and may not be generalizable to other PFA technologies. Fifth, CT-based anatomical measurements were performed by a single operator, and inter- and intra-observer reproducibility was not assessed. Finally, intracardiac echocardiography was not systematically used to independently confirm catheter-tissue contact and anatomical location. Although catheter contact and location were assessed using impedance-based contact sensing in combination with fluoroscopy and electroanatomic mapping, discrepancies between the mapped and actual catheter position cannot be completely excluded and may have contributed to unintended lesion formation. In addition, although map shifts were manually corrected, catheter location was verified fluoroscopically, and post-ablation maps were reconstructed *de novo*, residual registration error cannot be completely excluded.

## Patient’s perspective

The patients were informed that electroanatomic mapping identified unintended right atrial lesions after ablation. At the time of manuscript revision, all four patients had completed at least 3 months of follow-up. At the 3-month follow-up, all patients were in sinus rhythm on 12-lead electrocardiography, and none reported palpitations or other symptoms suggestive of recurrent atrial arrhythmia. No symptoms or clinical events attributable to the unintended right atrial lesions were observed during follow-up. They supported publication of these observations in the hope that the findings may contribute to improving the safety of future catheter ablation procedures.

## Lead author biography


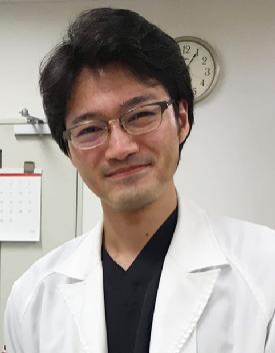


Jun Hirokami graduated from Sapporo Medical University in 2015 and completed his training in internal medicine, cardiology, and cardiac electrophysiology at Teine Keijinkai Hospital and Kokura Memorial Hospital, Japan. He subsequently served as an Electrophysiology Fellow at Cardioangiologisches Centrum Bethanien in Frankfurt, Germany. He is currently a staff cardiologist and electrophysiologist at Kokura Memorial Hospital. His research focuses on atrial fibrillation ablation, advanced mapping technologies, and pulsed-field ablation. He has authored more than 20 peer-reviewed publications, including several as first author in leading electrophysiology journals, and has received several research awards at national scientific meetings.

## Data Availability

The supporting data including images relating to the cases can be made available by the primary and corresponding authors on request.
